# Factors promoting or inhibiting normal birth

**DOI:** 10.1186/s12884-018-1871-5

**Published:** 2018-06-18

**Authors:** Samantha J. Prosser, Adrian G. Barnett, Yvette D. Miller

**Affiliations:** 1 School of Psychology, The University of Queensland, Brisbane, Australia; 2 School of Public Health & Social Work, Institute of Health and Biomedical Innovation, Queensland University of Technology, Kelvin Grove, Brisbane, QLD 4059 Australia

**Keywords:** Normal birth, Spontaneous labour, Midwifery-led care, Vaginal birth, Patient-reported data

## Abstract

**Background:**

In response to rising rates of medical intervention in birth, there has been increased international interest in promoting normal birth (without induction of labour, epidural/spinal/general anaesthesia, episiotomy, forceps/vacuum, or caesarean section). However, there is limited evidence for how best to achieve increased rates of normal birth. In this study we examined the role of modifiable and non-modifiable factors in experiencing a normal birth using retrospective, self-reported data.

**Methods:**

Women who gave birth over a four-month period in Queensland, Australia, were invited to complete a questionnaire about their preferences for and experiences of pregnancy, labour, birth, and postnatal care. Responses (*N* = 5840) were analysed using multiple logistic regression models to identify associations with four aspects of normal birth: onset of labour, use of anaesthesia, mode of birth, and use of episiotomy. The probability of normal birth was then estimated by combining these models.

**Results:**

Overall, 28.7% of women experienced a normal birth. Probability of a normal birth was reduced for women who were primiparous, had a history of caesarean, had a multiple pregnancy, were older, had a more advanced gestational age, experienced pregnancy-related health conditions (gestational diabetes, low-lying placenta, high blood pressure), had continuous electronic fetal monitoring during labour, and knew only some of their care providers for labour and birth. Women had a higher probability of normal birth if they lived outside major metropolitan areas, did not receive private obstetric care, had freedom of movement throughout labour, received continuity of care in labour and birth, did not have an augmented labour, or gave birth in a non-supine position.

**Conclusions:**

Our findings highlight several relevant modifiable factors including mobility, monitoring, and care provision during labour and birth, for increasing normal birth opportunity. An important step forward in promoting normal birth is increasing awareness of such relationships through patient involvement in informed decision-making and implementation of this evidence in care guidelines.

**Electronic supplementary material:**

The online version of this article (10.1186/s12884-018-1871-5) contains supplementary material, which is available to authorized users.

## Background

The term ‘normal birth’ in academic literature and health policy has more generally come to refer to a birth without, or with limited, clinical intervention. A 2007 consensus statement by the Maternity Care Working Party, *Making Normal Birth a Reality*, called for a standard definition of normal birth to increase confidence in the auditing and monitoring of practice trends [1]. The resulting definition described normal birth as an unassisted vaginal birth without induction of labour; epidural, spinal or general anaesthesia; or episiotomy [2]. Unlike some definitions, Werkmeister’s definition [2] is limited to the *process* of birth and does not extend to *outcomes* of birth such as vertex presentation and intact perineum.

The 1990s and 2000s saw a steady increase in rates of medical intervention during labour and birth across a number of developed countries [3]. While such procedures can be life-saving, they also bear risk to women [4–7] and their babies [8, 9] and thus should be limited to instances of medical necessity. There has been increasing international interest in promoting normal birth and progression towards less medicalised models of birth in response to the rising rate of intervention. In 2005, The Royal College of Midwives launched the *Campaign for Normal Birth* within the UK, now continued as part of the *Better Births Initiative* [10]. Care providers in Canada have published a *Joint Policy Statement on Normal Birth* to support, promote and protect normal birth for women [11]. In Australia, current maternity care reform (*National Maternity Services Plan: 2010*) [12] is based on an underlying philosophy of birth as a normal physiological event. Normal birth guidelines were published in the Australian state of Queensland in 2012 to protect, promote and support normal birth [13]. Additionally, the *Towards Normal Birth in New South Wales* policy directive required all birthing facilities in that Australian state to have a written policy for normal birth by 2015 [14].

Despite increasing international interest there is relatively limited evidence for how to best facilitate normal birth as a multi-dimensional construct. To date, very few studies have examined determinants of normal birth in this way (i.e., based on Werkmeister’s definition) [2]. The Birthplace in England national prospective cohort study [15] found that the rate of normal birth for low risk women differed depending on the place in which they were giving birth. Normal birth was more likely in freestanding midwifery units or at home than within obstetric units. More recently, Miller and colleagues [16] examined a wider range of predictors and reported that women were more likely to experience normal birth if they were in a public rather than private facility, did not have continuous fetal monitoring, were able to move freely during their labour, gave birth in a non-supine position, or delivered their baby outside standard business hours. Primiparous women were less likely to experience normal birth [16]. These studies identify a number of modifiable factors that can be used to inform practice aimed at promoting normal birth. However, the samples were limited to low risk women or women who had a vaginal birth, respectively, and findings may not generalise to all women (for example, as a means of preventing intrapartum caesarean or for women with obstetric risk factors).

Other research on factors that may reduce intervention during birth has focused on individual procedures, such as induction of labour or caesarean section. In regards to modifiable factors, characteristics of care are one source of variation. There is greater use of intervention in private birthing facilities than in public facilities, including induction of labour, epidural anaesthesia, episiotomy, instrumental vaginal birth, and caesarean section [17–21]. These differences are not attributable to case mix [22, 23]. Rates of intervention have also been shown to increase for women outside midwifery-led continuity models of care [24], who receive care from a larger number of nurses in labour [25], give birth during business hours [26, 27], or have continuous electronic fetal monitoring during labour [28]. More specifically, some practices have been associated with reduced need for regional anaesthesia during labour, such as water immersion during first stage labour [29], upright positioning and freedom of movement [30] and continuous one-to-one support [31]. There is some consistency between these findings and those directly evaluating associations with normal birth as an outcome.

Less modifiable characteristics have also been associated with increased intervention during birth. Rates of intervention are typically increased for women with obstetric conditions such as gestational diabetes, pre-eclampsia or placenta praevia [18, 32, 33]. While not a risk factor in and of itself, primiparity is linked to higher rates of induction [32, 34], epidural usage [35], and caesarean section [36]. Furthermore, women with a history of caesarean section are at a high likelihood of having a repeat caesarean for this reason only [37]. Women with a body mass index (BMI) in the ‘obese’ range are at greater odds having an induced labour [32] and a caesarean section [18, 38, 39] and older maternal age is often associated greater use of intervention [40]. However, the relationship between age and intervention is not always consistent, may be indirect, and may depend on whether women have given birth previously [41].

The aim of this study was to expand current evidence for factors which may promote or inhibit women achieving a normal birth. We examined a range of both modifiable (e.g. continuity of care) and non-modifiable (e.g. parity) factors, using secondary analysis of retrospective, self-reported, population-level survey data of women’s maternity care experience. Additionally, we aimed to expand on the methods used in previous studies by providing the probabilities of achieving normal birth as a singular construct among all women, irrespective of their risk status.

## Method

### Background/sampling

All women who gave birth in Queensland, Australia, across a 4-month period (October 2011 to January 2012, inclusive) were invited by the Queensland Registry of Births, Deaths and Marriages to complete the 2012 Having a Baby in Queensland Survey; a cross-sectional evaluation of women’s experiences of pregnancy, labour, birth, and postnatal care [42]. Women who experienced a stillbirth or neonatal death were sent a tailored survey and not included in the current study. Women received the survey 3 to 4 months after birth and could complete it either online, on paper (returned using a provided reply-paid envelope), or over the telephone with a trained female interviewer and a translator if required. A reminder/thank you postcard was sent to all women approximately two weeks after the initial mail-out. This survey and all subsequent analyses received clearance from The University of Queensland’s *Behavioural & Social Sciences Ethical Review Committee*. Completion of the anonymous survey was taken as evidence of consent to participate.

For women who had a live birth, the usable response rate was 30.4% (5840 out of 19,194). The respondent sample was approximately representative of the 2011 birthing population of Queensland for mode of birth, previous caesarean section, plurality of pregnancy, area of residence, infant birthweight and gestational age at birth [43, 44]. The sample under-represented younger women (aged less than 20 years), Aboriginal and/or Torres Strait Islander women, and women birthing in public facilities. Respondent characteristics are compared with those of the 2011 Queensland birthing population in Additional file 1.

### Outcome variables

Normal birth was defined according to the Werkmeister and colleagues definition [2]. Women were categorised as having experienced a normal birth if they did not have an induction of labour, epidural, spinal and/or general anaesthesia, an assisted vaginal birth (with forceps and/or ventouse) or caesarean section, or an episiotomy. Normal birth was broken into four categorical time-dependent outcomes: onset of labour, anaesthesia, mode of birth, and episiotomy. This was to allow the inclusion of variables that may not have been relevant for all women (e.g. water immersion in labour which was only relevant for women who experienced labour). For each subsequent outcome variable, only women who were still eligible for meeting normal birth criteria were included. For example, only those who had a spontaneous onset of labour were included in analyses examining use of anaesthesia during labour.

Onset of labour was assessed through a series of questions about whether women had experienced medical or surgical procedures to induce labour, and whether women had experienced any labour at all. Women were coded as having a spontaneous labour if they did not have prostaglandins, artificial rupture of membranes, synthetic oxytocin, or a balloon catheter for the purposes of inducing labour. Women who had a spontaneous labour were coded according to whether they used anaesthesia during labour using the item ‘*Did you have an epidural or spinal (anaesthetic injection in your back) for pain relief during labour?*’. For women who did not use anaesthesia during labour we assessed mode of birth by asking ‘*How was your baby born?*’ with five response options: *an unassisted vaginal birth*, *a vaginal birth assisted with a vacuum*, *a vaginal birth assisted with forceps*, *a vaginal birth assisted by forceps and a vacuum*, *a caesarean birth*. Women who had an unassisted vaginal birth were then further categorised using the item, ‘*During your birth, did you have an episiotomy (cut with scissors or a scalpel) to enlarge your vaginal opening?*’, according to whether they had an episiotomy. The remaining women who did not have an episiotomy were those who had a normal birth.

### Independent variables

Based on previous literature we selected a range of independent variables, including demographic and obstetric characteristics, antenatal and intrapartum care, experiences during labour and birth, and organisational factors. These variables are detailed in Additional file 2.

### Statistical models

Multiple logistic regression models were used to estimate the associations between the independent variables and the four binary outcomes: onset of labour, anaesthesia, mode of birth, and episiotomy. Results are presented as probability ratios. As a simple example, if the probability of a normal birth was 0.2 for women in the reference group and 0.1 in another group then the probability ratio would be 0.5 (0.1 divided by 0.2), meaning that the probability was halved compared with the reference group. These probability ratios are used instead of odds ratios (the ratios of odds) which are often less intuitive and prone to misinterpretation – particularly with highly prevalent outcomes.

To estimate the probability of a normal birth we multiplied the estimated probabilities from the four logistic models (the probabilities of a spontaneous onset of labour, no anaesthesia, an unassisted vaginal birth, and no episiotomy). For example, if a woman had a 0.80 probability at each of the four stages than her probability of a normal birth would be 0.80^4^ = 0.41.

We used a Bayesian paradigm to fit the logistic models so that we could multiply together the four probabilities and create a mean and 95% credible interval for the probability of normal birth. Ninety-five per cent credible intervals are like 95% confidence intervals but have the simpler interpretation of having a 95% probability of containing the true value.

Relationships between the independent variables were checked for co-linearity using variance inflation factors. Type of facility (public or private) and place of birth were found to have too much overlap with model of care, and were excluded for this reason. For water immersion during birth there was perfect prediction, with *all* women birthing in water experiencing a normal birth. This variable was excluded from the analysis as it is likely a proxy for an intervention-free birth rather than a predictor. Continuous variables were centred and scaled to help with the interpretation of the regression models.

Analyses were conducted using R version 3.0.2 (2013–09-25) software combined with JAGS to run the models [45, 46]. We used a burn-in of 10,000 MCMC iterations followed by 10,000 samples thinned by 3.

## Results

Of the total sample, 53.1% had a spontaneous labour, 47.5% birthed without an epidural, spinal or general anaesthesia, 53.7% had an unassisted vaginal birth and 53.2% gave birth vaginally without an episiotomy. Combining these four outcomes, the overall rate of normal birth was 28.7% and was 44.3% among women who had a vaginal birth. Descriptive information relative to the independent variables is provided in Additional file 3.

### Probability of having spontaneous labour

Women had higher probability of experiencing spontaneous labour if they had completed secondary education. Relative to private obstetric care, women had a higher probability of having a spontaneous labour if they were receiving GP shared care, standard public care, public midwifery continuity care, or private midwifery care.

The probability of experiencing spontaneous labour was lower for women with gestational diabetes, high blood pressure, other risk factors, or a multiple pregnancy. Women who were primiparous, multiparous with a history of caesarean, older, of higher BMI, or of later gestational age were also at a lower probability of having a spontaneous labour. The mean probability ratios and 95% credible intervals are presented in Table 1.

**Table 1:** Estimated mean probability ratios and 95% credible intervals of spontaneous labour, no anaesthesia, unassisted vaginal birth and no episiotomy

	Probability Ratios							
	Spontaneous Labour		No Anaesthesia		Unassisted VB		No Episiotomy	
	(*N* = 4611)		(*N* = 2223)		(*N* = 1636)		(*N* = 1322)	
	M	95% CI	M	95% CI	M	95% CI	M	95% CI
MATERNAL SOCIO-DEMOGRAPHIC CHARACTERISTICS								
Maternal age (+ 5 years)^a^	0.94	0.91–0.98	0.90	0.82–0.98	0.99	0.97–1.00	0.96	0.87–1.00
Completed secondary education	1.21	1.06–1.38	1.21	0.87–1.69	0.98	0.94–1.02	0.94	0.75–1.15
Identifies as Aboriginal/Torres Strait Islander	1.01	0.70–1.33	0.94	0.56–2.31	0.91	0.69–1.00	0.80	0.27–1.08
Area of residence								
Major city	1.00		1.00		1.00		1.00	
Inner regional	1.05	0.96–1.14	1.31	1.05–1.69	1.00	0.98–1.02	1.02	0.94–1.15
Outer regional	1.06	0.97–1.16	1.75	1.33–2.44	1.00	0.97–1.02	1.05	0.98–1.22
Remote/very remote	0.94	0.74–1.16	1.59	0.91–3.86	1.03	1.00–1.08	1.01	0.77–1.23
PREGNANCY DETAILS/COMPLICATIONS								
Parity								
Multiparous – no previous CS	1.00		1.00		1.00		1.00	
Multiparous – previous CS	0.38	0.32–0.45	0.64	0.50–0.82	0.55	0.28–0.81	0.72	0.32–0.98
Primiparous	0.81	0.74–0.88	0.53	0.41–0.68	0.79	0.59–0.93	0.66	0.32–0.93
Multiple birth	0.47	0.28–0.70	0.50	0.32–0.87	0.06	0.00–0.56	–	
Pre-pregnancy BMI (+ 5 kg/m^2^)^b^	0.96	0.93–0.98	1.00	0.96–1.05	1.00	1.00–1.01	1.02	1.00–1.09
Gestational age (+ 1 week)^c^	0.95	0.92–0.97	0.95	0.91–0.99	1.00	0.99–1.00	0.97	0.91–1.00
Gestational diabetes	0.70	0.59–0.82	0.94	0.70–1.35	0.97	0.90–1.01	1.03	0.87–1.25
High blood pressure	0.51	0.42–0.60	0.73	0.57–0.96	1.02	0.99–1.06	0.95	0.72–1.07
Low lying placenta	0.88	0.76–1.00	0.80	0.62–1.05	0.98	0.92–1.01	1.02	0.87–1.19
Other risk factors	0.76	0.69–0.82	1.15	0.97–1.40	1.00	0.97–1.01	1.00	0.91–1.08
ANTENATAL AND INTRAPARTUM CARE								
Model of care								
Private obstetric care	1.00		1.00		1.00		1.00	
Standard public care	1.40	1.29–1.54	1.35	1.07–1.77	1.00	0.97–1.02	1.09	1.01–1.83
GP shared care	1.48	1.35–1.62	1.67	1.32–2.24	1.02	1.00–1.05	1.08	1.00–1.32
Public midwifery continuity care	1.66	1.50–1.86	1.57	1.20–2.18	1.02	1.00–1.07	1.07	1.00–1.31
Private midwifery care	1.93	1.64–2.25	3.35	0.96–109.68	1.03	0.98–1.09	1.14	1.01–1.58
Known care providers – labour/birth								
None of them	1.00		1.00		1.00		1.00	
Some of them	–		0.76	0.63–0.91	0.95	0.87–0.98	0.96	0.83–1.03
All of them	–		1.36	0.84–2.76	1.01	0.97–1.06	1.07	0.95–1.34
Continuity of care during labour/birth	–		1.45	1.21–1.78	1.02	1.00–1.05	1.03	0.98–1.16
Felt rushed/hurried during labour	–		1.19	0.92–1.60	0.94	0.84–0.99	1.09	1.00–1.38
LABOUR/BIRTH EXPERIENCE								
Augmentation of labour								
Yes	1.00		1.00		1.00		1.00	
No	–		2.72	2.15–3.56	1.01	0.99–1.03	1.05	1.00–1.19
Not sure	–		1.31	0.85–2.36	0.93	0.78–1.00	0.91	0.52–1.12
Had continuous fetal monitoring	–		0.47	0.35–0.63	0.92	0.82–0.98	0.95	0.80–1.02
Freedom of movement throughout labour	–		1.54	1.20–2.01	1.02	1.00–1.06	1.08	0.99–1.35
Water immersion during labour	–		1.05	0.85–1.36	1.00	0.98–1.02	1.03	0.96–1.16
Birth outside business hours	–		–		1.01	0.99–1.03	1.01	0.93–1.10
Non-supine position during birth	–		–		–		1.11	1.01–1.41

*Note*. *M* Mean probability ratio, *CI* Credible interval, *CS* Caesarean section, *VB* Vaginal birth

^a^Age centred at 30 years

^b^BMI centred at 28 kg/m^2^

^**c**^Centred at 39 weeks gestation

### Probability of not using Anaesthesia

The probability of not using anaesthesia during labour was higher for women living in inner and outer regional areas, receiving GP shared care, standard public care, or public midwifery continuity care, not receiving augmentation of labour, experiencing freedom of movement throughout labour, or who had continuity of carer for labour/birth (see Table 1).

Women had a lower probability of not using anaesthesia if they were: primiparous, multiparous with a history of caesarean, older, of a later gestational age at birth, having a multiple birth, experiencing high blood pressure, having continuous electronic fetal monitoring during labour, or receiving care from only some known care providers during labour and birth.

### Probability of unassisted vaginal birth

The probability of having an unassisted vaginal birth was reduced for women who were: primiparous, multiparous with a history of caesarean, having a multiple birth, having continuous electronic fetal monitoring, felt rushed during their labour, or received their labour and birth care from only some known care providers (see Table 1).

### Probability of not having an episiotomy

Women had a higher probability of avoiding episiotomy if they received standard public care or private midwifery care, or if they gave birth in a non-supine position. The probability of avoiding episiotomy was lower for primiparous women and multiparous women with a previous caesarean section. Mean probability ratios and 95% credible intervals are presented in Table 1.

### Probability of normal birth

The probabilities of the four outcomes were multiplied to give the overall probability of normal birth for each independent variable (see Table 2). Overall, the probability of having a normal birth was higher for women: living in inner and outer regional areas; receiving GP shared care, standard public care, public midwifery continuity care, or private midwifery care; who had freedom of movement throughout labour; received continuity of care in labour and birth; who had no augmentation of labour; and who gave birth in a non-supine position.

**Table 2:** Estimated mean probability ratios and 95% credible intervals of a normal birth

	Probability Ratios for Normal Birth	
	Mean	95% CI
MATERNAL SOCIO-DEMOGRAPHIC CHARACTERISTICS		
Maternal age (+ 5 years)^a^	0.84	0.74–0.92
Completed secondary education	1.25	0.92–1.70
Identifies as Aboriginal and/or Torres Strait Islander	0.71	0.20–1.29
Area of residence		
Major city	1.00	
Inner regional	1.22	1.03–1.46
Outer regional	1.39	1.16–1.74
Remote/very remote	1.19	0.77–1.70
PREGNANCY DETAILS/COMPLICATIONS		
Parity		
Multiparous – no previous CS	1.00	
Multiparous – previous CS	0.10	0.03–0.20
Primiparous	0.22	0.09–0.37
Multiple birth	0.01	0.00–0.12
Pre-pregnancy BMI (+ 5 kg/m^2^)^b^	0.98	0.93–1.05
Gestational age (+ 1 week)^c^	0.90	0.83–0.94
Gestational diabetes	0.68	0.48–0.91
High blood pressure	0.39	0.26–0.53
Low lying placenta	0.76	0.55–0.98
Other risk factors	0.81	0.70–0.94
ANTENATAL AND INTRAPARTUM CARE		
Model of care		
Private obstetric care	1.00	
Standard public care	1.77	1.46–2.31
GP shared care	2.00	1.64–2.61
Public midwifery continuity care	2.22	1.81–2.83
Private midwifery care	3.19	2.03–4.93
Known care providers – labour/birth		
None of them	1.00	
Some of them	0.75	0.61–0.88
All of them	1.26	0.93–1.71
Continuity of care during labour/birth	1.24	1.09–1.48
Felt rushed/hurried during labour	1.12	0.92–1.45
LABOUR/BIRTH EXPERIENCE		
Augmentation of labour		
Yes	1.00	
No	1.44	1.22–1.80
Not sure	0.96	0.51–1.32
Had continuous fetal monitoring	0.34	0.24–0.46
Freedom of movement throughout labour	1.33	1.11–1.77
Water immersion during labour	1.06	0.90–1.26
Birth outside business hours	1.01	0.93–1.11
Non-supine position during birth	1.11	1.01–1.41

Note**.** ^a^Age centred at 30 years; ^b^BMI centred at 28 kg/m^2^; ^c^Centred at 39 weeks gestation

The probability of having a normal birth was reduced for women who were primiparous, multiparous with a previous caesarean, older, had a multiple pregnancy, had a later gestational age, had gestational diabetes, had a low-lying placenta, had high blood pressure, experienced other risk factors, had continuous electronic fetal monitoring in labour, and had only some known care providers for labour and birth.

## Discussion

This study aimed to identify factors which may facilitate or impede normal birth, using the definition of Werkmeister and colleagues [2] where normal birth is an unassisted vaginal birth without induction of labour, epidural or general anaesthesia, or episiotomy. We calculated the probability of normal birth based on a range of modifiable and non-modifiable characteristics with retrospective, self-reported data. We found that less than half of women birthing vaginally (44.3%) and less than one-third of all birthing women (28.7%) experienced a normal birth. While consistent with previous Australian research [16], this rate is lower than estimated rates of normal birth (as defined by Werkmeister and colleagues) in Scotland (35.5%) [47] and England (42%) [48].

We identified several modifiable and non-modifiable factors that can inhibit or promote normal birth. In regards to factors that were non-modifiable at the time of pregnancy, women were less likely to experience normal birth if they were older, were primiparous, had previously given birth by caesarean, were having a multiple birth, had obstetric health concerns (including high blood pressure, low-lying placenta, gestational diabetes, or other risk factors), were at an earlier gestational age, or lived in a major city relative to an inner or outer regional area. These characteristics align with findings from previous studies of normal birth [16] and the broader literature around risk factors for medical intervention in birth [18, 32, 41].

Perhaps of greater clinical utility in the promotion of normal birth are the modifiable factors (after accounting for clinical risk) we found to change women’s likelihood of normal birth. The probability of normal birth was higher for women who received care in a model other than private obstetric care, had continuity of care during labour and birth, knew none rather than some of their care providers, had freedom of movement during labour, did not have continuous fetal monitoring or augmentation, or birthed in a non-supine position. Despite the sample and methodological differences in this study, these findings are largely consistent with those of Miller and colleagues [16].

The higher rate of obstetric intervention in private facilities is well-established [17–21]. By using model of care rather than place of birth, we allowed exploration of potential variation among different types of public care models. Relative to private obstetric care, all other models resulted in increased likelihood of normal birth for women. Women receiving GP shared care or public midwifery continuity care were at least twice as likely to have a normal birth, while it was three times higher for women in private midwifery care. Private obstetric care is the primary model of care in private facilities and is based around a medicalised view of birth. This contrasts with a more naturalist philosophy typically adopted in both public and private midwifery-led models [49]. These philosophical differences, along with the differing clinical skills of lead care providers, are likely to contribute to the disparate probabilities of normal birth for women receiving care in these models. Risk alone cannot account for the higher rate of intervention in private hospitals as discrepancies in outcomes remain after accounting for obstetric and social risk factors [18]. The relationship between model of care and normal birth is relevant for women’s decision-making about their care, however the associated risks and benefits of different models are infrequently discussed at the time this choice is made [50].

We also found that normal birth was less likely when women knew some rather than none of their care providers. It is possible that this may relate to differences in the organisation of labour and birth care between private and public models of care (and thus, private and public birth facilities). As we have demonstrated, models of care provided in public facilities (GP shared care, public midwifery continuity care and standard public care) increase the probability of achieving a normal birth, and may be less likely to provide a known care provider at the time of labour and birth care. However, having previously known care providers for labour and birth may be less relevant than having continuity of carer throughout this time. We found that women with continuity of carer for labour and birth (after accounting for known care providers and model of care) were 24% more likely to have a normal birth. Having continuous support during labour is argued to provide emotional support and comfort, information and advocacy, enhancing women’s sense of control and reducing the need for medical intervention [31]. While continuity of care across pregnancy, birth and beyond may be the ideal, having continuity of carer for labour and birth may be a cost-effective solution to reducing intervention where resources are not available for full continuity.

Our findings for a greater probability of normal birth among women who didn’t have continuous electronic fetal monitoring, had freedom of movement, or who birthed in a non-supine position are consistent with previous studies about normal birth [16]. Other research has identified that upright positioning during the first stage of labour can reduce the use of epidural anaesthesia [30] and the incidence of instrumental delivery [51], both factors that prevent a normal birth. Despite calls that the risks and benefits of fetal monitoring during labour should be discussed [52, 53], many women report that they are either not informed or not involved in decisions about its use [54]. The extent to which upright positioning and mobility during labour and birth are encouraged by care providers remains unclear.

In contrast to a previous study of normal birth [16], we did not identify timing of birth to be associated with the probability of experiencing normal birth. Rates of episiotomy [26] and instrumental vaginal birth [55] are shown to be higher during regular working hours, while the declining rate of weekend births appears to be related to the increasing rate of caesarean section [27]. Our lack of an association is potentially due to the stage at which timing of birth was introduced in the analysis. We did not examine timing of birth as a predictor among women who had an induction of labour or a planned caesarean – interventions where the effect of staff availability and scheduling logistics are likely to play a greater role in timing of birth. We also did not identify a relationship between water immersion during labour and normal birth. Aligned with our findings, a review of water immersion during the first stage of labour also found no effect on rates of assisted vaginal birth or caesarean section [29]. However, we also did not replicate the association between water immersion and the reduced need for epidural or spinal anaesthesia identified in this review [29]. Our measurement of water immersion during labour did not account for duration, which may result in a less consistent relationship. There is currently a lack of evidence and need for further research around the optimal conditions for water immersion during labour and how factors such as duration, depth of immersion, temperature and mobility may affect this.

A strength of this study is that it appears to be the first to have examined probabilities of normal birth as a multi-dimensional construct for a wide range of predictors and with a non-restricted sample of women. The study also used population-level sampling to invite participation and was uniquely able to use a combination of clinical and experiential factors due to the self-reported nature of the data, which has not been possible using clinical reporting alone. A high level of concordance between maternal self-report and medical records has been repeatedly demonstrated for clinical information pertaining to birth [56–58].

While the respondent sample was representative of the Queensland birthing population on many key clinical characteristics, some characteristics were under-represented. We could have used sampling weights to provide a closer match to the Queensland birthing population; however such weights are generally more important for estimating prevalence, whereas our study was concerned with the association between variables which we assumed were generalisable from our sample to the wider population. We also did not examine the inter-relationships between different predictors of normal birth. While we identified certain groups of women at a reduced likelihood of achieving a normal birth, such as primiparous women or those with a history of caesarean section, we did not examine how the chances of normal birth may be increased for such groups. Future research may wish to consider potential interactions between modifiable and non-modifiable characteristics to determine how intervention could be reduced among particular groups of women.

## Conclusions

Despite increasing interest in normal birth, actual rates remain low and research for how to facilitate this is minimal. Models of care with a more natural philosophy of birth, not limiting women’s freedom of movement and position during labour and birth, and continuity of care provider throughout labour and birth are shown to increase the likelihood of achieving a normal birth. To support the desired promotion of normal birth, care providers and women must be made aware of existing evidence for how care and treatment related factors influence normal birth outcomes. Pragmatic evaluation research is needed for how policies that relate to facilitating factors affect women’s experience of normal birth.

## Additional files

Additional file 1: Comparison of Respondent Sample and Population Characteristics. This file contains two tables comparing maternal and infant characteristics of the sample population with those of the Queensland, Australia, birthing population in 2011. (PDF 642 kb)

Additional file 2: Measurement of independent variables examined for associations with normal birth. This file contains a table detailing the independent variables used in the analysis, describing how they were measured in the Having a Baby in Queensland 2012 Survey. (PDF 484 kb)

Additional file 3: Sample characteristics of women as frequencies and percentages. This file presents a table outlining the characteristics of women in the study sample. Means and standard deviations, or frequencies and percentages where relevant, are provided. (PDF 486 kb)

## Abbreviations

BMI: Body mass index
CI: Credible intervals
CS: Caesarean section
GP: General practitioner
