# Supplementary material for: Factors promoting or inhibiting normal birth
Source: BMC Pregnancy Childbirth. 2018 Jun 18;18:241. doi: 10.1186/s12884-018-1871-5 (PMC6006773; doi:10.1186/s12884-018-1871-5)
Supplement: Supplementary file 1 — Comparison of Respondent Sample and Population Characteristics. This file contains two tables comparing maternal and infant characteristics of the sample population with those of the Queensland, Australia, birthing population in 2011. (PDF 642 kb) [file 12884_2018_1871_MOESM1_ESM.pdf]

Additional File 1. Comparison of Respondent Sample and Population Characteristics

Table 1. Maternal characteristics: Comparison of survey respondents and women who gave birth in Queensland in 2011.

Maternal Characteristics	Survey Sample (N=5,840)		Queensland Population 2011 (N=61,125)[1]	
	N	%	N	%
Maternal Age in Years				
Less than 20	104	1.9	3,120	5.1
20-24	638	11.5	10,307	16.9
25-29	1,639	29.4	17,835	29.2
30-34	1,920	34.5	17,688	28.9
35-39	1,039	18.6	9,955	16.3
40 and over	232	4.2	2,220	3.6
Not stated	*268*		0	
Indigenous Identification				
Aboriginal or Torres Strait Islander	89	1.6	3,646	6.0
Non-Indigenous	5,536	98.4	57,453	94.0
Not stated	*215*		*13*	
Area of Residence[2]				
Major city	3,514	62.5	37,134	61.3
Inner regional	1,059	18.8	11,529	19.0
Outer regional	845	15.0	9,407	15.5
Remote & very remote	151	2.7	2,522	4.2
Not applicable/Outside QLD	55	1.0	n/a	
Not stated	*216*		*512*	
Parity				
Primipara	2,640	46.1	25,132	41.1
Multipara	3,083	53.9	35,993	58.9
Not stated	*117*		*0*	
Pre-pregnancy BMI[3]				
Underweight (BMI <18.50)	275	5.2	3,161	5.3
Normal (BMI 18.50 - 24.99)	3,005	57.1	30,394	50.6
Overweight (BMI 25.00 - 29.99)	1,132	21.5	14,745	24.6
Obese (BMI ≥30.00)	854	16.2	11,758	19.6
Not stated	*574*		*1,054*	
Maternal Country of Birth				
Australia	4,522	80.0	47,093	77.0
Outside Australia	1,127	20.0	14,032	23.0
Not stated	*191*		*0*	

[1] Based on Queensland Perinatal Statistics report for 2011 births (n=61,125).
Queensland Health. Perinatal statistics: Queensland 2011. Brisbane, QLD: Queensland Department of Health; 2014.
[2] Figures for the Queensland population sample sourced from Australia's Mothers & Babies Report (n=61,112).
Li Z, Zeki R, Hilder L, Sullivan EA. Australia's mothers and babies 2011. Perinatal statistics series no. 28. Cat. no. PER 59. Canberra: AIHW National Perinatal Epidemiology and Statistics Unit; 2013.
[3] Figures from Australia's Mothers & Babies Report (n=61,112).

Maternal Characteristics	Survey Sample (N=5,840)		Queensland Population 2011 (N=61,125)[1]	
	N	%	N	%
Place of Birth				
Public Hospital	3,335	57.1	41,901	68.5
Private Hospital	2,282	39.1	17,885	29.3
Birth Centre	161	2.8	841	1.4
Home	32	0.5	69	0.1
Other	30	0.5	429	0.7
Facility Type				
Public	3,496	59.9	42,742	69.9
Private	2,282	39.1	17,885	29.3
Home births	32	0.5	69	0.1
Other	30	0.5	429	0.7
Onset of Labour[2]				
Spontaneous	3,043	53.1	34,335	56.2
No augmentation	1,694	29.6	22,030	36.0
Medical augmentation	275	4.8	3,800	6.2
Surgical augmentation	723	12.6	6,455	10.6
Combined augmentation	245	4.3	2,033	3.3
Other/not stated	106	1.9	17	0.0
Induced	1,461	25.5	14,180	23.2
Medical only	635	11.1	4,729	7.7
Surgical only	165	2.9	1,135	1.9
Combined	653	11.4	8,170	13.4
Other/not stated	8	0.1	146	0.2
No labour	1,222	21.3	12,595	20.6
Not stated	*114*		*2*	
Method of Birth[4]				
Non-instrumental vaginal	3,095	53.7	34,933	57.2
Forceps	548	1.7	1,142	1.9
Vacuum	96	9.5	4,743	7.8
Forceps and vacuum	55	1.0	n/a	-
Caesarean – with labour	743	12.9	7,698	12.6
Caesarean – no labour	1,222	21.2	12,595	20.6
Not stated	*81*		*1*	
Previous Caesarean Birth[5]				
None	2,084	69.1	25,282	70.2
At least one	932	30.9	10,711	29.8
Not stated	*67*		*0*	
Plurality of Pregnancy				
Singleton	5,742	98.3	60,098	98.3
Twin	94	1.6	1,001	1.6
Other multiple	4	0.1	24	0.0
Not stated	*0*		*2*	

[4] Figures from Australia's Mothers & Babies Report (n=61,112)
[5] Multiparous women only

Table 2. Infant characteristics: Comparison of survey respondents' infants and infants born in Queensland in 2011

Infant Characteristics	Survey Sample (N=5,942)		Queensland Population 2011 (N=61,780)[6]	
	N	%	N	%
Infant Gestational Age in Weeks				
Less than 28	21	0.4	325	0.5
28-36	492	8.4	4,801	7.8
37-41	5,147	88.4	56,299	91.1
42 or more	164	2.8	349	0.6
Not stated	*118*		*6*	
Infant Birth Weight in Grams				
Less than 1,500	42	0.7	696	1.1
1,500-2,499	322	5.6	3,286	5.3
2,500-4,499	5,193	90.9	56,683	91.8
4,500 and over	159	2.8	1,111	1.8
Not stated	*226*		*4*	

[6] Figures for the Queensland population were sourced from the Queensland Health Perinatal Statistics report and were based on live singleton and multiple births in 2011.
