# Supplementary material for: Factors promoting or inhibiting normal birth
Source: BMC Pregnancy Childbirth. 2018 Jun 18;18:241. doi: 10.1186/s12884-018-1871-5 (PMC6006773; doi:10.1186/s12884-018-1871-5)
Supplement: Supplementary file 2 — Measurement of independent variables examined for associations with normal birth. This file contains a table detailing the independent variables used in the analysis, describing how they were measured in the Having a Baby in Queensland 2012 Survey. (PDF 484 kb) [file 12884_2018_1871_MOESM2_ESM.pdf]

Additional File 2. Measurement of independent variables examined for associations with normal birth.

Variable	Measurement	Categories/Scale
MATERNAL SOCIO-DEMOGRAPHIC CHARACTERISTICS		
Maternal age	Maternal age at the time of birth, calculated from mother's date of birth and baby's date of birth.	Continuous. Centred at 30 years.
Secondary education	'*What is the highest level of qualification you have completed?*'. Those indicating '*Year 12 or equivalent*', '*Trade/apprenticeship*', '*Certificate/Diploma*', or any university-level education, were coded as having 'Completed' secondary education. Those indicating '*Year 10 or equivalent*' or '*No formal qualifications*' were coded as having 'Not completed' secondary education.	Completed/Not completed
Aboriginal and/or Torres Strait Islander identification	'Yes' if indicated being either '*Aboriginal*' or '*Torres Strait Islander* '. 'No' if '*None of the above'* was selected.	Yes/No
Area of residence	Derived from suburb/town and postcode of women's usual place of residence at the time her baby was born. Categories created based on the Australian Standard Geographical Classification system (ABS, 2010).	Major city; Inner regional; Outer regional; Remote/Very remote
PREGNANCY DETAILS/COMPLICATIONS		
Parity	Women were asked separately how many times they had been pregnant, had given birth, and had had a caesarean section. Women who indicated one pregnancy or one birth were coded as '*Primiparous*'. Women indicating two or more births were coded as '*Multiparous*'. Multiparous women were further divided into whether they had previously had a caesarean.	Multiparous – no previous caesarean section; Multiparous – previous caesarean section; Primiparous
Plurality	Women received tailored questionnaires based on whether there had been a single or multiple birth registrations on the Registry of Births, Deaths and Marriages' database.	Single/Multiple
Pre-pregnancy BMI	Calculated as weight (kg)/height(m)^2 from reported pre-pregnancy weight and height.	Continuous. Centred at 28 (kg/m^2)
Gestational age	Women were asked to indicate '*How many weeks pregnant were you when your baby was born?*' in weeks and days.	Continuous. Centred at 39 weeks

Variable	Measurement	Categories/Scale
Gestational diabetes	'*During your pregnancy, did a care provider tell you that…You had gestational diabetes (diabetes due to pregnancy)?*'	Yes/No
High blood pressure	'*During your pregnancy, did a care provider tell you that…You had high blood pressure (hypertension, pre-eclampsia)?*'	Yes/No
Low lying placenta	'*During your pregnancy, did a care provider tell you that…You had placenta praevia (placenta close to or covering your cervix)?*'	Yes/No
Other risk factors	Women were coded as having experienced any other risk factors if they answered 'yes' to any of the following: '*During your pregnancy, did a care provider tell you that… You were experiencing depression?...You were experiencing anxiety?... Your amount of amniotic fluid ('waters') was a concern?...You had a problem with your cervix?... There was a problem with your baby's cord?... Your baby was too big?... Your baby was too small?... Your membranes had ruptured (waters had broken) and labour did not start?...Other?*'. Where other specified risk factors were accounted for by the variables described above (i.e. Previous caesarean, multiple pregnancy, maternal age, maternal BMI, gestational diabetes, placenta praevia/low lying placenta, pregnancy-induced hypertension/pre-eclampsia or parity), women were coded as 'No' for having additional risk factors.	Yes/No
ANTENATAL AND INTRAPARTUM CARE		
Model of care	Women were asked, '*What type of pregnancy and labour/birth care did you have?*' and asked to select from a list of options. Responses were than cross-checked against information such as place of birth, continuity of midwifery care, and intrapartum transfer, and reclassified where applicable.	Private obstetric care; Standard public care; GP shared care; Public midwifery continuity care; Private midwifery care
Known care providers for labour/birth	Women were asked if they had met their care providers for labour/birth before they went into labour. Response options were '*All of them*', '*Some of them*', and '*None of them*'.	None of them; Some of them; All of them
Continuity of carer for labour/birth	'*Was there at least one maternity care provider who cared for you right through your labour and birth?*'	Yes/No
Rushed/hurried during labour	*'Did you feel rushed or hurried by your care provider(s) at any time during your labour?'*	Yes/No

Variable	Measurement	Categories/Scale
LABOUR/BIRTH EXPERIENCE		
Continuous fetal monitoring during labour	In response to '*During your labour, how was your baby monitored (checked)?*, women indicating '*A monitor was used constantly with a belt around my stomach*' or '*A monitor was used constantly with a clip on my baby's head*' were coded as 'Yes' for having experienced continuous monitoring. Remaining women indicating '*A Doppler (hand held monitor) was used occasionally*', '*Staff listened with a stethoscope (or ear trumpet) occasionally*', '*A monitor was occasionally with a belt around my stomach*', or '*My baby was not monitored*' were coded as 'No'. Open-text responses were back-coded into the relevant category. Responses of '*Not sure if/how my baby was monitored*' treated as missing data for this item.	Yes/No
Augmentation of labour	Women who indicated that a care provider ruptured their membranes or that they had a Syntocinon infusion, for the purpose of augmenting (or 'speeding up') their labour, were coding as 'yes'. Women who reported having neither procedure were coded as 'no' and were who were unsure were coded as 'not sure'.	Yes; No; Not sure
Freedom of movement throughout labour	Women were asked, '*During your labour, were you able to move around and choose the position that made you most comfortable?*'. Response options of '*No*', '*Yes, some of the time*', and '*Yes, most of the time*' were coded as 'no' for not having freedom of movement 'all of the time' and responses of '*Yes, all of the time*' were coded as 'yes'.	Yes/No
Water immersion during labour	'*Did you use a pool or bath in your place of birth for pain relief during labour?*'	Yes/No
Timing of birth	Date and time women gave birth used to derive a measure of whether a birth occurred in 'business hours'. Births occurring between the hours of 08:00 and 17:00, from Monday to Friday, coded as 'Inside business hours'. Births occurring on weekends or outside the hours of 08:00 and 17:00 on weekdays coded as 'Outside business hours'.	Inside business hours/Outside business hours
Position during birth	Women were asked '*What was the final position you were in when your baby was born?*'. Response options of '*Lying on my back (stirrups or no stirrups)*' or '*Semi sitting (stirrups or no stirrups)*' were coded as 'Supine'. Responses of '*Lying on my side*', '*Sitting on a birth stool*', '*Hands and knees*', '*Kneeling*', '*Sitting*', '*Standing*', '*Squatting*', or '*Other*', were coded as 'Non-Supine'. Drawings accompanied each position to aid interpretation.	Supine/Non-supine

Note. For women with a multiple pregnancy, where relevant, responses in relation to the birth of their first baby were used.
