# Supplementary material for: Factors promoting or inhibiting normal birth
Source: BMC Pregnancy Childbirth. 2018 Jun 18;18:241. doi: 10.1186/s12884-018-1871-5 (PMC6006773; doi:10.1186/s12884-018-1871-5)
Supplement: Supplementary file 3 — Sample characteristics of women as frequencies and percentages. This file presents a table outlining the characteristics of women in the study sample. Means and standard deviations, or frequencies and percentages where relevant, are provided. (PDF 486 kb) [file 12884_2018_1871_MOESM3_ESM.pdf]

Additional File 3. Sample characteristics of women as frequencies and percentages

	Total Sample (N=5,840) ^	
	N/Mean	%/(SD)
MATERNAL SOCIO-DEMOGRAPHIC CHARACTERISTICS		
Maternal age in years	30.53	(5.29)
Secondary education		
Not completed	491	8.7
Completed	5,162	91.3
Aboriginal and/or Torres Strait Islander identification		
No	5,536	98.4
Yes	89	1.6
Area of residence		
Major city	3,514	63.1
Inner regional	1,059	19.0
Outer regional	845	15.2
Remote/very remote	151	2.7
PREGNANCY DETAILS/COMPLICATIONS		
Parity		
Multiparous – no previous caesarean	2,084	36.8
Multiparous – previous caesarean	932	16.5
Primiparous	2,640	46.7
Plurality		
Single	5,742	98.3
Multiple	98	1.7
Pre-pregnancy BMI *(kg/m^2)*		
Underweight (<18.5)	275	5.2
Normal (18.5-25.0)	3,005	57.1
Overweight (25.0-30.0)	1,132	21.5
Obese (>30.0)	854	16.2
Gestational age in weeks	39.25	(2.02)
Gestational diabetes		
No	5,233	91.5
Yes	484	8.5
High blood pressure		
No	5,084	89.1
Yes	624	10.9
Low lying placenta		
No	5,229	91.6
Yes	478	8.4
Other risk factors		
No	3,482	60.7
Yes	2,255	39.3
ANTENATAL AND INTRAPARTUM CARE		
Model of care		
Private obstetric care	2,355	43.9
Standard public care	1,070	19.9
GP shared care	1,224	22.8
Public midwifery continuity care	684	12.7
Private midwifery care	37	0.7
Known care providers – labour/birth		
None of them	1,896	33.1
Some of them	3,434	60.0
All of them	391	6.8

	Total Sample (N=5,840) ^	
	N/Mean	%/(SD)
Continuity of care – labour/birth		
No	1,592	27.8
Yes	4,131	72.2
Rushed/hurried during labour		
No	3,837	89.9
Yes	430	10.1
LABOUR/BIRTH EXPERIENCE		
Continuous fetal monitoring		
No	1,997	47.8
Yes	2,177	52.2
Augmentation of labour		
Yes	1,764	30.8
No	3,798	66.2
Not sure	172	3.0
Freedom of movement throughout labour		
No	2,306	53.6
Yes	1,994	46.4
Water immersion – labour		
No	3,693	86.3
Yes	588	13.7
Time of birth		
Inside business hours	2,241	38.4
Outside business hours	3,599	61.6
Position during birth		
Supine	2,674	70.9
Non-supine	1,097	29.1

SD = Standard deviation; Frequencies may not total 5,840 due to missing data or questions being not applicable for some women.
